# A collaborative guide to Rapid Invisible Frequency Tagging (RIFT): Methods, insights, and recommendations

**DOI:** 10.1162/IMAG.a.1273

**Published:** 2026-06-17

**Authors:** Kabir Arora, Cecília Hustá, Floortje Bouwkamp, Noor Seijdel, Songyun Bai, Qiu Han, J. Leon Kenemans, Stefan Van der Stigchel, Surya Gayet, Eelke Spaak, Samson Chota, Linda Drijvers

**Affiliations:** Helmholtz Institute, Utrecht University, Utrecht, The Netherlands; Max Planck Institute for Psycholinguistics, Nijmegen, The Netherlands; Donders Institute for Brain, Cognition and Behaviour, Radboud University, Nijmegen, The Netherlands

**Keywords:** RIFT, SSVEP, neural oscillations

## Abstract

Rapid Invisible Frequency Tagging (RIFT) is a recent advance in frequency tagging that exploits novel, high-frequency displays to modulate luminance at imperceptibly high frequencies. RIFT goes beyond low-frequency tagging by allowing researchers to track neural responses to rhythmic stimulation while avoiding perceptual confounds. RIFT is thus a promising method to address central questions in rhythmic cognition, including attention, multimodal integration, and the neural mechanisms underlying oscillatory coordination in perception. However, setting up a RIFT study involves several technical and conceptual considerations. In an effort to make RIFT more accessible, we provide a comprehensive guide for implementing RIFT in cognitive neuroscience. On the basis of the joint experiences and data-driven insights of multiple laboratories, we provide practical recommendations derived from empirical datasets to improve reproducibility, covering hardware requirements, stimulus design, analysis approaches, and interpretation of results. We hope that this guide helps readers to both identify the conceptual areas where RIFT offers promising insights and navigate the technical caveats that come with the approach.

## 1 Introduction

Rhythms are omnipresent in our brain and environment, and shape how we attend to, perceive, and integrate information. In cognitive neuroscience, a fundamental challenge lies in the way we measure how rhythmic neural activity relates to ongoing cognitive processing without interfering with perception itself.

Frequency tagging has been a widely used stimulus presentation method within the field of cognitive neuroscience (Norcia et al., 2015). In short, it involves varying a specific property of a stimulus (e.g., luminance) at a fixed frequency. This rhythmic modulation induces a neural response at the same frequency, called Steady-State Evoked Potentials (SSEPs). Because this response is frequency specific, it provides a powerful marker of how the brain processes the tagged stimulus over time. Frequency tagging has, therefore, been widely used to study attention (e.g., spatial attention (Morgan et al., 1996; Müller & Hübner, 2002), feature-based attention (Müller et al., 2006; Pei et al., 2002), perceptual selection, and multimodal integration (Alp et al., 2018; Regan & Regan, 1988)).

**Fig. 1.**
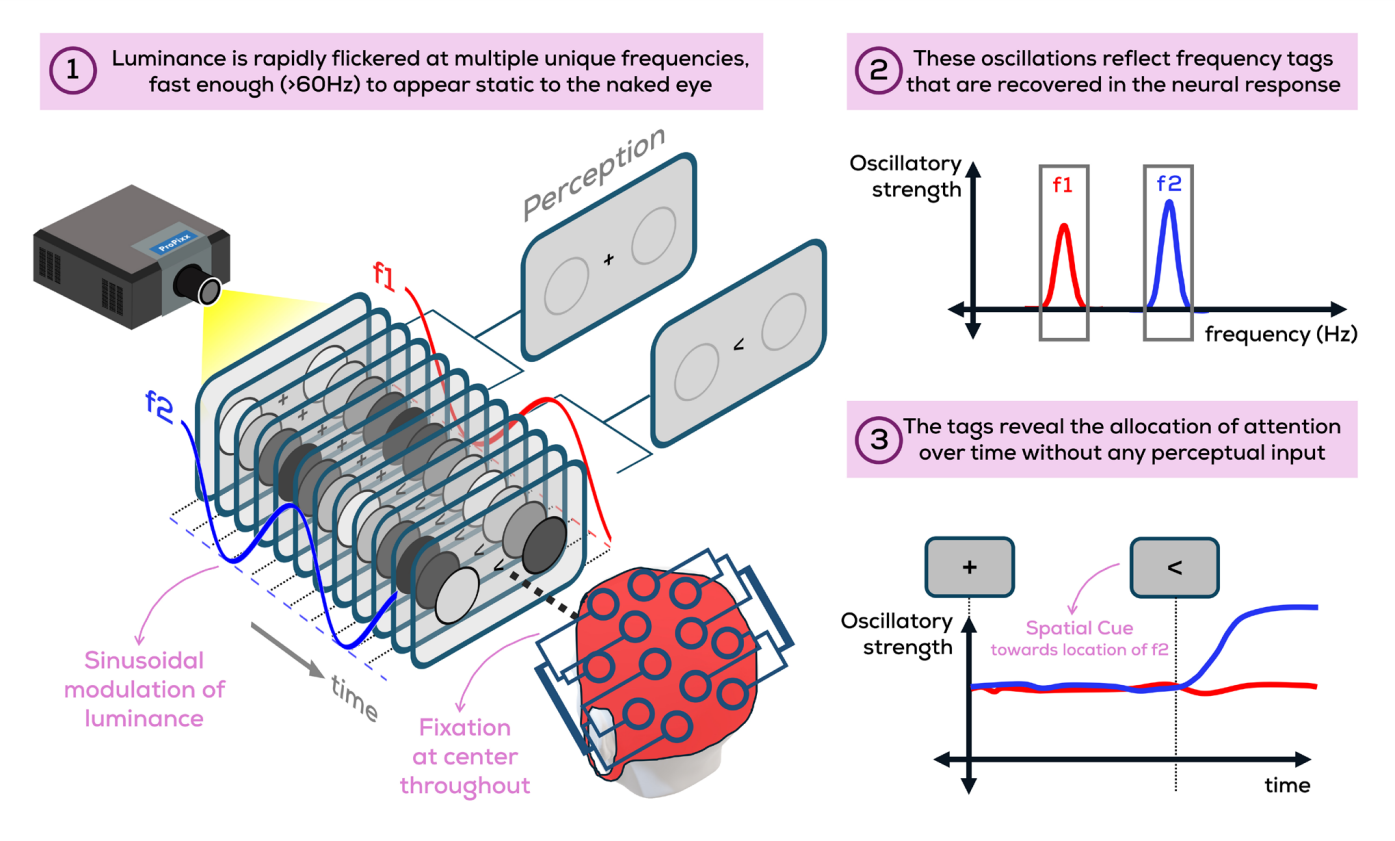
Rapid invisible frequency tagging. The luminance of stimuli or areas on the screen is modulated sinusoidally at frequencies that exceed the threshold of perception. The response to these tags can then be uniquely recovered in the M/EEG response, and its amplitude over time can reveal location or feature-based modulations in covert attention. Thus, RIFT forms a non-invasive, continuous tracker of attention in the absence of any visible probes.

Recent advances in display technology have given rise to an innovative new branch of frequency tagging: Rapid Invisible Frequency Tagging (RIFT). Unlike low-frequency tagging, which typically flickers stimuli at frequencies below 30 Hz, RIFT flickers stimuli at frequencies higher than 60 Hz (Seijdel et al., 2023; Zhigalov et al., 2019), allowing the tagging to become virtually imperceptible (Fig. 1). RIFT shares the benefits that SSEPs already offer, such as the ability to separate neural responses to multiple simultaneous stimuli. RIFT additionally provides several advantages over existing SSEP applications. Firstly, at lower frequencies, luminance changes are visible. This makes them easily identifiable, and they may automatically draw attention (Cass et al., 2011). RIFT, by flickering complex stimuli beyond the threshold of visibility, offers a tracker of visual processing without perceptual interference. This results in more naturalistic paradigms where the spatial spread of attention can be continually measured without any awareness of this probe. Another major benefit is that RIFT can measure the allocation of attention to locations which appear indistinguishable from background space. In doing so, it offers a qualitatively different tool for studying relatively difficult-to-measure cognitive processes that occur in the absence of any stimuli. Lastly, in the low-frequency range (<30 Hz), SSEP responses may be difficult to disentangle from endogenous oscillations in similar frequency bands, or may even entrain or disrupt them (Notbohm et al., 2016; Spaak et al., 2014). The RIFT response does not interact with endogenous oscillations: its typical frequencies are far away from lower bands such as alpha (Arora, Gayet, et al., 2025; Gutteling et al., 2022; Zhigalov & Jensen, 2020), and even spectrally close oscillations such as gamma are not entrained (Duecker et al., 2021). Though LEDs have been used to investigate the periodic neural response to high-frequency flickers in the past (Gulbinaite et al., 2019; Herrmann, 2001), only recently has technology allowed for the display of complex stimuli at high flicker frequencies, with the term “RIFT” reflecting the integration of high-frequency flicker with more regular cognitive experiments.

RIFT is essentially a tracker of visual processing, used most frequently as an invisible tracker of attention. Given the large range of cognitive phenomena that involve any variation in visual processing or attentional deployment, whether over space, time, or both, RIFT has the potential to produce novel insights for a variety of cognitive fields. For example, RIFT has been shown to be a powerful tool to capture covert shifts of attention both to tagged stimuli (Zhigalov et al., 2019) and to tagged, but perceptually indistinguishable regions of visual space (i.e., tagging the background; Arora, Gayet, et al., 2025). Over the last few years, RIFT has been applied within the domains of reading (Pan et al., 2021, 2024), distractor suppression (Ferrante et al., 2023), visual search (Bouwkamp et al., 2025; Duecker et al., 2025), visual working memory (Arora, Gayet, et al., 2025), temporal processing (Dietz et al., 2025), brain–computer interfacing (Brickwedde et al., 2022), multimodal integration (Drijvers et al., 2021; Seijdel et al., 2024), and the interaction between speech planning and comprehension representations (Hustá et al., 2025). Aside from exploring novel cognitive contexts that may benefit from using the technique, current RIFT research is also focused on technical aspects such as optimizing display features (Minarik et al., 2023) and exploring alternative forms of tagging (Spaak et al., 2024).

In sum, RIFT has proven to be a sensitive method for tracking neural responses and their modulation by cognitive demands, and previous work shows clear and promising potential for RIFT within cognitive research. However, the method also introduces novel technical and analytical considerations that are not yet widely documented.

In this perspective, we consolidate the joint experiences and data-driven insights of multiple independent laboratories to provide a comprehensive guide with resources and recommendations for running a RIFT study. We describe best practices for (1) hardware requirements, (2) experimental design and stimulus presentation, and (3) analysis of the neural response. This manual primarily compiles previously undocumented knowledge acquired through the setup and operation of new RIFT laboratories, as well as through in-depth exploration of technical aspects of previously collected RIFT datasets. Our goal is to make RIFT more accessible to researchers across various fields, and to highlight how RIFT can contribute to the study of rhythmic cognition across domains.

## 2 Hardware Considerations

### 2.1 Why does RIFT require devices with fast refresh rates?

An important feature of RIFT is that visual stimuli are tagged above the threshold of perceptibility (>60 Hz). Achieving this requires specialized display tools with high-speed refresh rates that go beyond the refresh rates available in standard monitors.

**Fig. 2.**
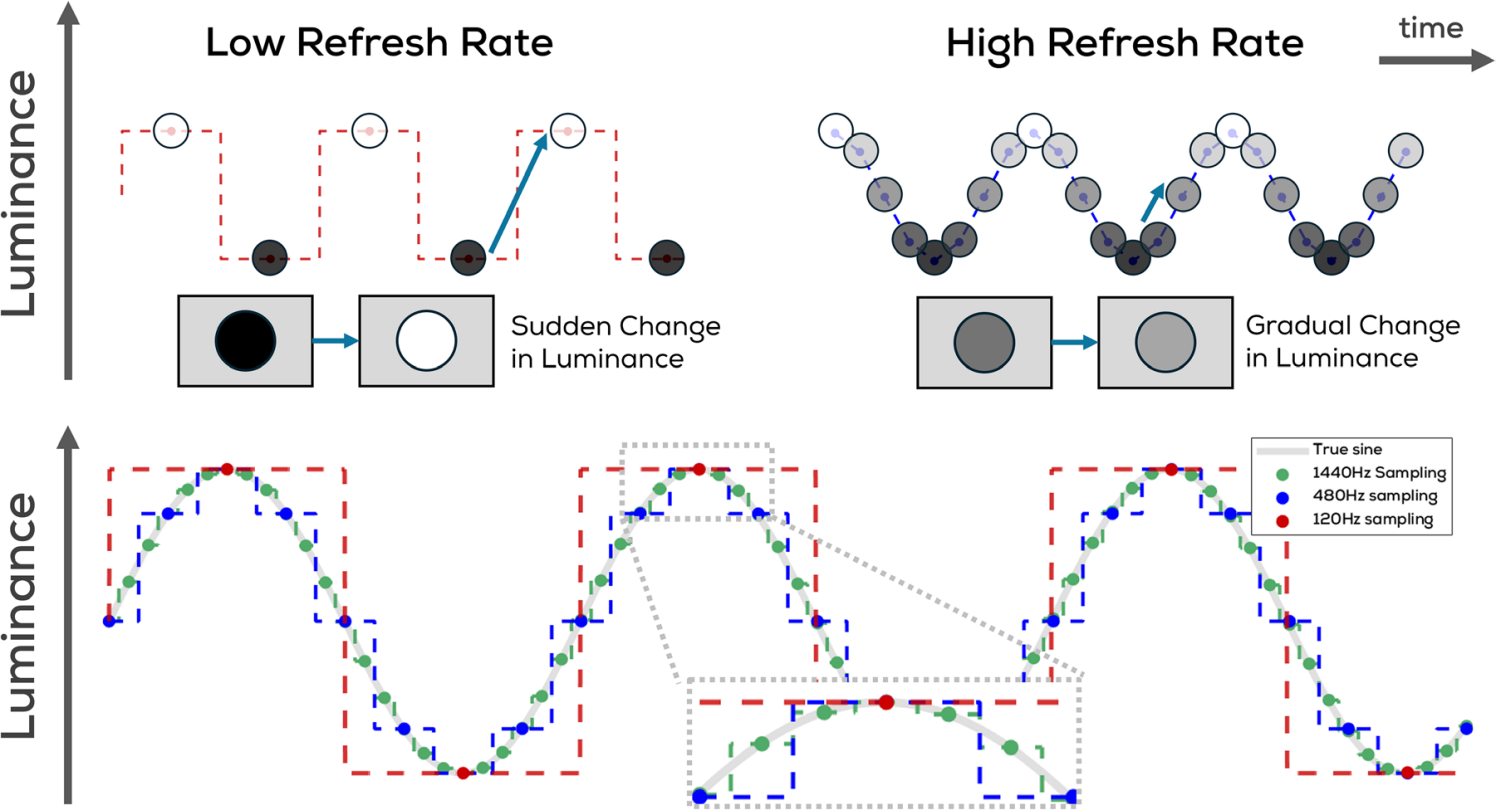
Higher refresh rates more closely reflect sinusoidal modulations of luminance.

Conventional monitors, typically running at up to 120 Hz, are ill-suited for several reasons. Firstly, a 120 Hz monitor can *only* produce a 60 Hz RIFT tag, by alternating between black and white on each frame. However, given that RIFT is mainly used as a tracker of spatial attention, most cognitive questions using RIFT depend on contrasting the attentional resources dedicated to different locations in the same visual environment. Thus, RIFT studies often use two or more high-frequency tags simultaneously, for example, 60 Hz and 64 Hz (Arora, Gayet, et al., 2025), or 60 Hz, 64 Hz, and 68 Hz (Bouwkamp et al., 2025), which a 120 Hz monitor is not capable of doing. Secondly, the interval between two consecutive frames is quite short. Standard monitors rarely have the ability to shift through their full luminance range (black – minimum, to white – maximum) during this short interval. Our own personal experiments demonstrated that standard monitors have trouble doing so, leading to visible tagging modulations on the screen. Though the tagging could also use a lower luminance range (e.g., flicker from grey to black) if a monitor is incapable of using its full luminance range quickly, this has been shown to weaken the tagging response (Spaak et al., 2024). Lastly, even if perfect control of luminance at 120 Hz is achieved, there is a stark difference between the resulting step-function luminance trace achievable at a 120 Hz refresh rate and the sine-approaching traces achievable at higher refresh rates. The latter avoid sudden and large jumps in luminance (Fig. 2), resulting in much reduced visibility of the luminance oscillation. Indeed, it has been shown that, when using 120 Hz refresh rates, even 60 Hz oscillations are visible (Waldin et al., 2017). However, with rapid refresh rates (i.e., 1440 Hz), the 60 Hz tag has been experimentally shown to be invisible (Spaak et al., 2024).

### 2.2 Which devices can accomplish this?

Nearly all RIFT research so far has made use of a PROPixx DLP projector (VPixx technologies), which supports refresh rates up to 1440 Hz (if using greyscale) or 480 Hz (if colour is used). PROPixx circumvents the bandwidth limitations of graphics cards by repackaging multiple frames into each input image (see Box 1), allowing for imperceptible rhythmic stimulation at a precision that is unmatched by standard hardware. However, given the expenses associated with this option, it is worth exploring viable alternatives. More recently, newer commercially available OLED monitors operating at 480 Hz have emerged as a viable alternative, by using fast pixel response times, which increases accessibility (Dimigen et al., 2025). Early evidence exists that demonstrates that these consumer-grade monitors produce robust RIFT responses. We, therefore, believe that as the use of RIFT becomes more widespread, this monitor option will soon be more utilized. Thus, our discussions and suggestions below are general to any device and refresh rate (with the exception of Box 2).

BOX 1. WHAT IS LUMINANCE TAGGING?

As mentioned above, RIFT involves the sinusoidal modulation of some visual feature of an object, or an area of the visual field, at a fixed frequency. This feature is most commonly luminance. Here, we briefly explain the idea of luminance tagging in more detail before exploring its applications and features in Sections 2 and 3.

Flickering a stimulus entails alternating the luminance with which it is presented between dark and bright values. So, tagging a circular patch involves cycles of briefly presenting a dark circle, followed by a bright circle, over and over. The duration of this luminance cycle is determined by the desired frequency of tagging. For a 60 Hz tag (60 cycles a second), each cycle lasts 1/60 = 0.0166 seconds. The actual luminance levels that are used depend on the refresh rate of the device used. The higher the device’s refresh rate, the more luminance increments can be used in a cycle. As more and more luminance levels are included with higher refresh rates, luminance traces are typically chosen to minimize the size of the increments, resulting in a sinusoidal tagging trace (Fig. 2). This is because the use of small frame-to-frame luminance changes contributes to the invisibility of RIFT, compared with non-sinusoidal (e.g., black-to-white) flicker modulations, typical in slower SSVEP research.

BOX 2. HOW DOES THE PROPIXX PROJECTOR ACHIEVE HIGHER-THAN-GPU REFRESH RATES?

The core limitation overcome by the ProPixx projector is not merely high-speed display, since LED displays can also display rapidly changing visual luminance. In order to display complex stimuli, the display tool must be paired with a graphics card that can produce frame-by-frame images quickly enough to keep up with a fast display rate. The ProPixx takes away this requirement. That is, the graphics card being used can simply operate as if it was connected to a simple 120 Hz refresh rate device, but the projector is able to convert this input to a much faster refresh rate (480 Hz if using colour, 1440 Hz if not using colour).

**Fig. 3.**
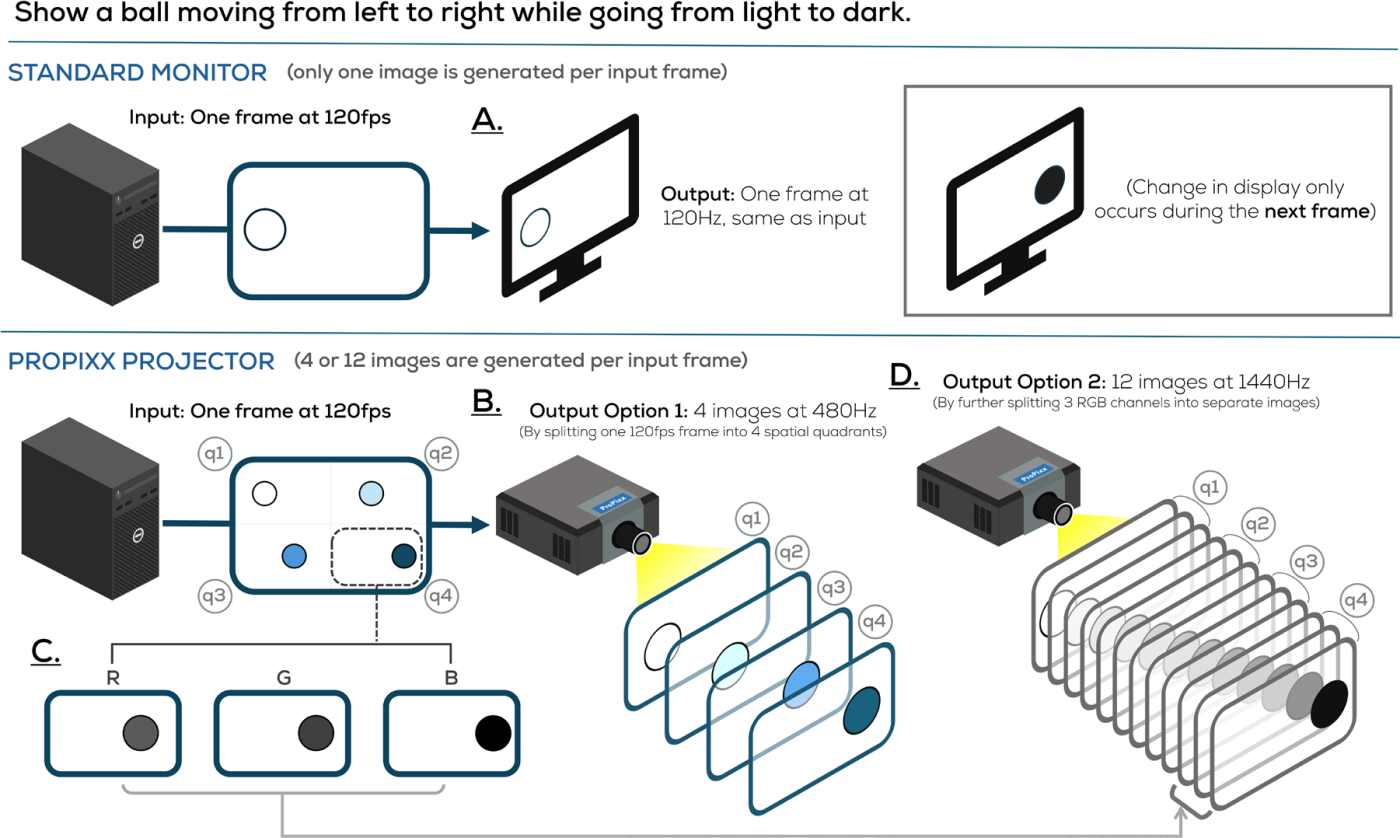
Operation of the PROPixx projector. When connected to a 120fps PC input, (A) a standard monitor displays one image for each frame. In the same time, the PROPixx projector can display either (B) 4 images by splitting up the frame into quadrants and displaying them sequentially, producing a 480 Hz output, or, (C) 12 images by further splitting the R, G, and B colour channels of the frame to produce 3 images per each of the 4 quadrants, (D) resulting in a 1440 Hz framerate. Note that the input from the PC has to be encoded specifically to produce the desired output after quadrant splitting and RGB splitting.

The ProPixx achieves this by allowing the user to “pre-package” up to 12 frames worth of information within a single image. For a normal monitor/PC setup, the graphics card sends out a frame which the monitor then displays. Thus, for a 120 Hz monitor setup, 120 images are displayed per second (Fig. 3A). The projector is able to achieve a higher refresh rate with the same input speed (120 images per second) from the graphics card. First, it divides the image received from the graphics card into four equal quadrants. Each of these quadrants is then sequentially displayed (Fig. 3B). This quadruples how many images the projector can display per frame sent by the graphics card resulting in a 480 Hz refresh rate. Note that this reduces the available screen resolution (from 1920 x 1080 to 960 x 540). The quadrant image separation means that any image which is intended to be drawn at one point on the screen must actually be drawn at four different locations (the centres of each of the four “quadrants”) which are offset in different directions from the true centre of the image. Here we include code (quadifier.m) compatible with MATLAB Psychtoolbox that carries out this operation by converting coordinate information from a screen-centred reference to a quadrant-centred reference.

This refresh rate can then further be tripled to achieve a 1440 Hz refresh rate for greyscale images. For this, the three colour channels (red, green, and blue) that normally add up to produce one colour image can be used to transmit three unique images (Fig. 3C) which are then displayed in sequence. In this way, the projector can display three frames per quadrant, resulting in 12 total images per frame sent by the graphics card (Fig. 3D).

### 2.3 Components of a RIFT setup

The main hardware component of a RIFT setup is the high-refresh display device (see Section 2.2). Because of the low demands the PROpixx projector places on the graphics card itself (Box 2), the projector setup has relatively lenient graphics card requirements. Our setups utilize relatively inexpensive graphics cards (e.g., NVIDIA Quadro K620 2 GB, GeForce GTX960 2 GB, and Radeon RX570 8 GB). The GPU requirement is considerably higher when using a high-refresh rate monitor for RIFT instead of the PROpixx projector (Dimigen & Stein, 2024; Dimigen et al., 2025).

Irrespective of which device is used for RIFT, a photodiode (luminance sensor) is essential for identifying and verifying that the intended modulation is faithfully displayed. Even timing errors on a millisecond level can shift the phase of the frequency-tagged signal, undermining experimental precision. For example, with a 60 Hz tag (1 cycle = ~16.6 ms), an 8 ms delay would put the tagging signal in anti-phase, so even a 1 ms delay will have a notable impact, and thus accounting for these inaccuracies becomes critical.

## 3 Stimuli/Experimental Design Considerations

### 3.1 Which frequencies are best to use for tagging?

#### 3.1.1 Theoretical range

With RIFT, we measure neural responses to imperceptible periodic stimulation. This definition leads to two automatic (theoretical) thresholds for RIFT frequencies. The first is a maximum frequency above which the response to periodic stimulation is no longer observed in the M/EEG signal. The second is a minimum frequency below which the flicker is visible. Previous work has used LED-based displays to investigate the periodic neural response to flickers in the 1–100 Hz range (Gulbinaite et al., 2019; Herrmann, 2001), from which a rough threshold of around 80 Hz emerges as an upper limit (Minarik et al., 2023). However, mean estimates of the critical flicker-fusion threshold tend to be close to 50 Hz but vary between 30 and 60 Hz depending on numerous individual factors and cognitive states (Haarlem et al., 2024). Thus, for visual tagging, the flicker appears visible to most people below 50 Hz. This allows us to set a (conservative) theoretical RIFT tagging range of 60–80 Hz in the visual domain. RIFT has also been implemented slightly below this range at 56 Hz (Brickwedde et al., 2022), and similar tagging protocols have been applied at even lower frequencies (41–45 Hz; Marshall et al., 2024), though here invisibility was not verified.

Beyond this range, there are a number of technical aspects to consider that produce a more limited set of frequency options in practice.

#### 3.1.2 Higher frequencies evoke lower responses

Even though all frequencies within the theoretically plausible range (~60–80 Hz) have been shown to evoke a periodic response, this response decreases in strength as the frequency increases (Gulbinaite et al., 2019; Herrmann, 2001). An upper limit of 72 Hz has been suggested for observing tagging in 90% of the participant pool, above which this percentage decreases quickly (Minarik et al., 2023).

#### 3.1.3 Stimulus size and eccentricity constraints

The rough upper limit of 72 Hz suggested above, however, may not always be applicable depending on tagging parameters. The tagging response also heavily depends on the size and eccentricity of the tagged area. Larger tags, tags closer to fixation, and tags in the lower visual hemifield evoke stronger RIFT responses. Whether a particular frequency will or will not show a consistent tagging response depends on a complex interplay with these parameters. For example, though 68.5 Hz did not evoke a tagging response with 6 dva diameter circular patch tagged at 6.3 dva eccentricity (Arora et al., 2024), the same frequency did evoke a consistent tagging response with a smaller tag (3 dva side length square) at fixation (Arora, Van der Stigchel, et al., 2025). This complex interplay has not yet been fully charted out. Effects of frequency, size, and eccentricity have been separately studied (Minarik et al., 2023), providing rough guidelines for these parameters. However, at the moment there are no comparisons where these parameters are cross-varied, and thus it is difficult to recommend specific size thresholds for specific tagging frequencies. In Table 1, we summarize all the tagging parameters (frequency, size, and eccentricity) that have so far been successfully used. We do recommend for any design with a novel combination of tagging parameters that experimenters use a small sample (~5) prior to running the experiment to test whether or not significant tagging responses, independently of the conditions of interest, are observed.

**Table 1.** Stimulus and tagging considerations.

Study	Topic	Tagged Stimuli	Refresh Rate (Hz)	Tag Type & Frequency (Hz)	Tag Duration (ms)	Stimuli (# / Size / Loc.)
Zhigalov et al., 2019	Attentional effect on cortical excitability	Photographs of faces and houses	1440	63, 78	1500	2 / – / 8.3° eccentricity, below midline
Zhigalov & Jensen, 2020	Alpha oscillations and spatial attention	Photographs of faces and houses	1440	60–70 (broadband tag)	2000	2 / 5.7° / 3.8° eccentricity, below midline
Drijvers et al., 2021	Multimodal integration	Auditory verbs, gesture video	1440	A: 61, V: 68, IM: 7	~2000 average	2 / 10° × 6.5° / Midline
Duecker et al., 2021	Entrainment of gamma	Background, circular moving gratings	1440	52–90 (2 Hz steps)	2000	1 / 2.62° / Centre
Gutteling et al., 2022	Attentional gating via alpha-band oscillations	Clear or degraded photographs of faces	1440	63, 70	2350 – 3350	2 / 8° / 7° eccentricity, below midline
Pan et al., 2021	Parafoveal processing	Background under the target word	1440	60	1000	1 / ~2–3° × 1° / Midline
Brickwedde et al., 2022	BCI control via covert attention	Section of background with grainy texture	1440	V: 56, 60	2000 (train); continuous segmented to 1000 (test)	2 / – / Bilateral below midline
Ferrante et al., 2023	Statistical learning of distractor location	Gabor patches	480	55–75 (broadband tag)	1500 (filler) + 300 (search)	2 / 6° × 6° / 4° eccentricity in all quadrants
Minarik et al., 2023	Optimal tag parameters examining: (1) tagging frequency, (2) stimulus size, (3) stimulus position	Background section	1440	66 – frequency varied for 1) between 60 and 100 (4 Hz steps)	1400 + tapers	1 / 10° / Centre – size varied for 2) 2–12° in 1° steps – pos. varied for 3) midline, above, or below
Bouwkamp et al., 2025	Predictive visual search	T + L distractors	1440	60, 64, 68	≤2500	3 / 2.5° × 2.5° / variable (within 11° × 7.5°)
Pan et al., 2024	Parafoveal semantic processing in reading	Background under the target word	1440	60	1000	1 / ~2–3° × 1° / Midline
Seijdel et al., 2024	Multimodal integration; attention	Auditory verbs, gestures	1440	A: 58, V-att: 65, V-unatt: 63, IM: 5, 7	~2000	3 / – / Midline
Spaak et al., 2024	Optimal tag parameters examining: (1) imperceptibility, (2) type of tagging, (3) accounting for phase shifts	Circular gratings	1440	60, 66	1200 or 1500	1–2 / 2°–4° / Midline, above, or below
Arora, Gayet, et al., 2025	Internal vs. External Attention	Colour gratings, background	480	60, 64	Continuous	2 / 6° / 6° eccentricity, 2° below midline
Husta et al., 2025	Planning speech during comprehension	Auditory nouns, pictures	1440	A: 54, V: 68, IM: 14	A: ~877, V: 1000	2 / 7.1° × 8.4° / Centre
Duecker et al., 2025	Guided visual search	T + L distractors	480	60, 67	≤4000	17 or 33 / 1° × 1° / variable (within 10° × 10°)
Dietz et al., 2025	Temporal expectations	Square-wave gratings	480	60	Whole trial (≤3800)	1 / 6° / Centre
Wang et al., 2026	Attentional capture	Geometrical shapes (singleton targets + distractors)	480	60, 64	1300	6 / 4.3° × 4.3° / 6° eccentricity, in circular shape
Dimigen et al., 2025	RIFT on consumer monitor	disc stimulus matching background	480 (on OLED monitor)	60, 64	10000	1 / 2.55° + tapers / Centre or Periphery at 12°
Duecker et al., 2025	Role of alpha in visual search	T + L distractors	480	60, 67	Variable	17 or 33 / – / –

Notes: A = auditory tagging; V = visual tagging; IM = intermodulation frequency. Where not specified, “–” indicates data not reported or not applicable. Tag location and stimulus size are reported in degrees of visual angle (dva).

#### 3.1.4 Sampling

**Fig. 4.**
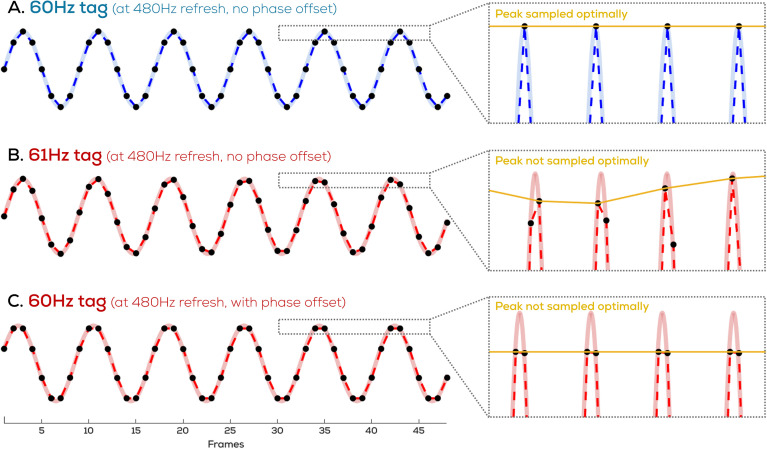
The choice of tagging frequency and phase can lead to presentation of imperfect sine waves. (A) When drawing a 60 Hz tag at a 480 Hz refresh rate, 8 (480/60) luminance points are equally sampled on each cycle and perfect sampling is observed if the phase is aligned to the sinusoid’s peaks. (B): When drawing a 61 Hz tag at the same refresh rate, every cycle does not have the same sampling of luminance points. (C) When drawing a 60 Hz tag at the same refresh rate but a different phase offset, the peaks are not sampled uniformly thus under-utilizing the dynamic range of the tagging.

When sinusoidally modulating luminance to display a tagged stimulus, the choice of tagging frequency and phase can lead to the presentation of imperfect sine waves, which can in turn result in dynamic luminance range being underutilized by the tagging. A “perfect” tag, that is, one that only stimulates the intended frequency without introducing any harmonics or other low-frequency components, would always capture the same values on the sinusoid for every cycle including the high and low peaks (e.g., Fig. 4A). This, however, is only possible when the tagging frequency is a factor of the refresh rate, in this example a 60 Hz (480 Hz / 8) tag. If the refresh rate is not an integer multiple of the tagging frequency, an artefact will arise because the peaks and troughs of the true sinusoid are not sampled regularly (e.g., Fig. 4B), in this example a 61 Hz (480 Hz / ~7.86) tag. Similarly, if the sinusoid being sampled has a phase offset such that no frames sample its peak or troughs, even a “perfect” tagging frequency can miss out on the full dynamic range of luminance (e.g., Fig. 4C). Such sampling issues can be avoided entirely by simply using frequencies that are an integer factor of the refresh rate (480 Hz/8 = 60 Hz, 480 Hz/7 = 68.57 Hz, 480 Hz/6 = 80 Hz) at the appropriate phase offset to ensure that the peaks of the sinusoid are sampled. This factor then equals how many points are sampled on each cycle, for example eight points per cycle for a 60 Hz tag. When using a 480 Hz refresh rate, this can result in a ~5% loss in dynamic range (See Supplementary Fig. S1 for an overview of how dynamic range varies with tagging frequency and refresh rate). Though experimental constraints may result in cases where it is preferable to sacrifice a small amount of dynamic range in order to use a larger number of unique frequency tags, this route offers a starting point for frequencies to select in the absence of any other limitations. These concerns are less pressing in cases of very high refresh rates, such as the 1440 Hz capabilities of a PROpixx projector. In these cases, losses in dynamic range and visible low-frequency aliasing artefacts are likely negligible (<0.5%).

#### 3.1.5 Power Line Noise

So far, 60 Hz has been a standard RIFT frequency given the technique’s prevalence in Europe. However, in North America, power line noise, which produces strong artefacts in M/EEG recordings, is also at 60 Hz. This makes the use of 60 Hz tagging (as well as other frequencies within the range of whichever filtering procedure is used to eliminate line noise) not feasible there. However, the wide range of frequencies used so far, as outlined in Table 1, combined with the use of phase-based tools such as coherence and phase skewing (see Sections 3.2.1 and 4.1.3), nonetheless confirms that RIFT can be implemented in these geographical areas.

### 3.2 What needs to be considered when tagging multiple stimuli?

RIFT studies commonly implement multiple tags to track visual processing of multiple stimuli or locations simultaneously. This involves some extra considerations.

#### 3.2.1 Spectral overlap

**Fig. 5.**
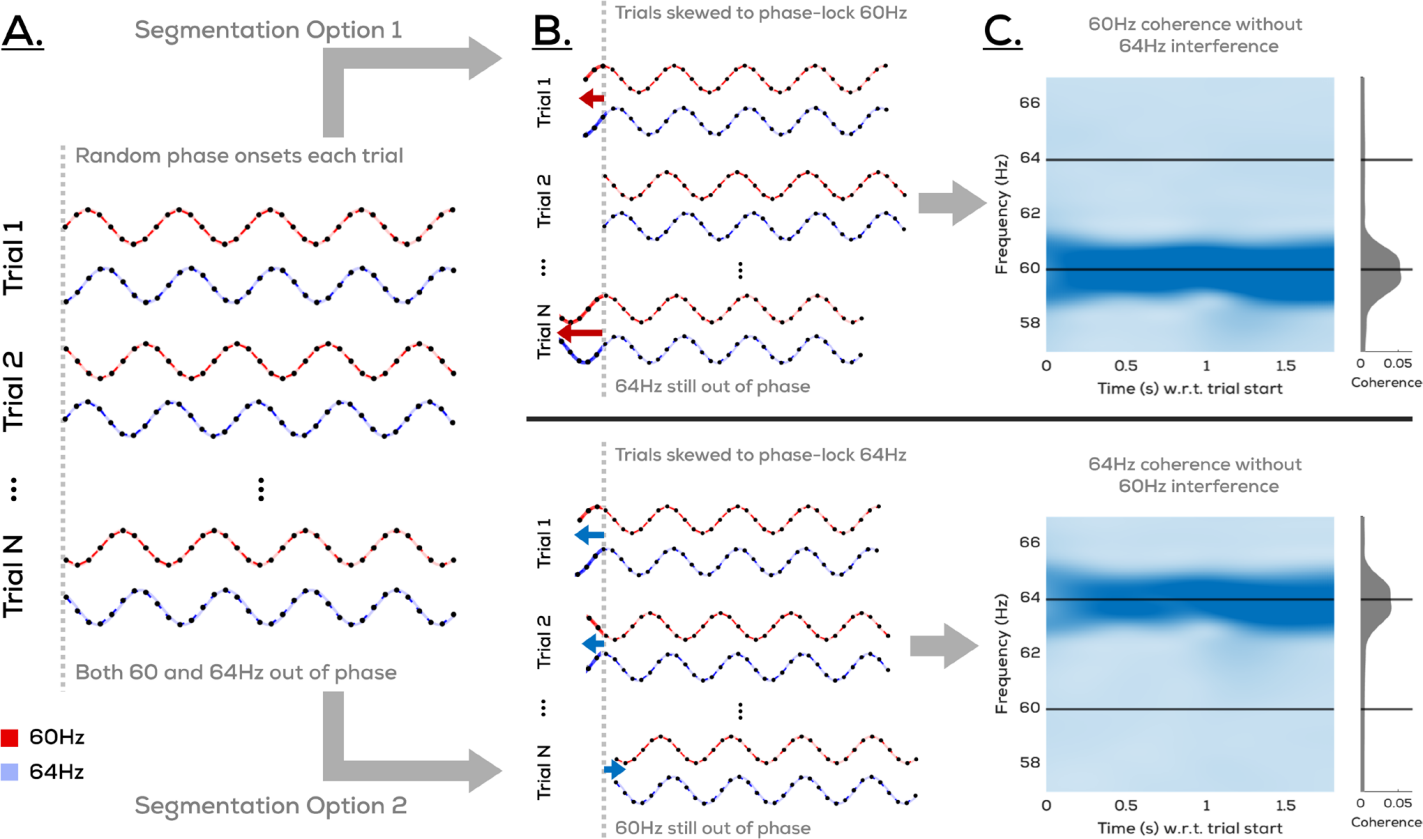
(A) When using multiple tags, temporal resolution is limited due to overlap effects. To maintain high temporal resolution, frequency resolution must be sacrificed. However, when using multiple tags, this is not possible beyond a limit due to overlap between the responses from both tags. (B–D) Phase can be used to avoid overlap effects across multiple tags. (B) To avoid these overlap effects, trials can be presented with tagging envelopes initialized at random phases. (C) Trials can then be separately analyzed by skewing to align the phases of one tag at a time. (D) This results in independent isolation of each tagging signal without the contribution of the other using coherence. Data averaged across 23 participants from Dataset 2.

Any time series analysis involves a trade-off between frequency resolution and temporal resolution: improving precision for one reduces precision for the other. For studies looking at RIFT modulations over time, it is ideal to have high temporal resolution (and thus low-frequency resolution). However, when tagging multiple stimuli at unique frequencies, frequency resolution cannot be too low because both tags must remain distinguishable. To illustrate this, we show how the mean discriminability of two frequency tags varies with parametrically modulated frequency resolution (Fig. 5A). Here, the periodic response is measured using filter-HT derived coherence (a technique to extract the amplitude of the oscillatory signal, explained further in Section 4.1). It is thus commonplace to select tags with wider separation in frequency space to optimize temporal resolution. This does decrease the similarity between both tags (higher frequencies evoke a lower tagging response), which is why it is essential to counterbalance different tags when using multiple frequencies (see Section 3.2.2).

It is possible to leverage tagging phase to increase the separability of different tags. Instead of maintaining a constant phase onset across trials that is also identical among different stimuli, the tagging signals can instead be offset by a random amount for each stimulus and each trial. Then, different tagging signals can later be separately analyzed from the same data without interference from one another (Fig. 5A) by epoching the data separately such that the epochs are phase locked with respect to only one particular signal at a time (Fig. 5B). All other tags will still remain scrambled in phase, and a measure such as coherence which relies on phase consistency will thus discount their contributions (Fig. 5C). To avoid variable trial lengths, either a slightly shorter trial window (~16 ms shorter for a 60 Hz tag) can be used post-skewing, or the trials can be zero padded to preserve the original trial window length. It is worth noting that using this procedure prevents the use of phase as a tagging information channel (see Section 3.5.1 for more about phase tagging).

#### 3.2.2 Counterbalancing

Counterbalancing tagging frequencies across participants—or, if possible, across trials—is important to avoid confounding frequency-specific effects with experimental conditions. Since higher frequencies elicit weaker neural responses, unique frequency tags are treated as different “channels” of information. To make meaningful comparisons across conditions, frequency–condition assignments can be balanced across participants, or better yet, within participants. Here, “condition” can refer to many feature dimensions such as stimulus type, location, and shape. Trial-level counterbalancing is especially powerful, as it controls for individual differences and increases sensitivity to condition-specific effects. However, it does reduce the available number of trials in each condition for measures such as coherence (see Section 4.1) which operate on a set of trials.

### 3.3 What needs to be considered when tagging greyscale vs. coloured stimuli?

When tagging a stimulus, its intensity is modulated sinusoidally; practically this involves multiplying some “luminance feature” of a stimulus with a time-varying sinusoidal envelope. Luminance can be tagged in various ways. For example, when tagging black and white gratings, it has been shown that modulating the white bands across the full luminance range (0–100%), or using contrast tagging (i.e., anti-phase flicker to white and black bands), produces a stronger response than other alternatives (Spaak et al., 2024). Furthermore, we have seen that frequency tagging a white luminance mask (modulating from 0% to 100% opaque) on top of object stimuli yields robust tagging responses for the corresponding stimuli, whereas modulating stimulus opacity directly, fading them in and out against a grey or textured background, results in markedly weaker signals (unpublished data).

Most design tools for psychophysics experiments directly use RGB colour codes when drawing stimuli. If a greyscale object is tagged, it is most straightforward to use this RGB colour code as the luminance feature that is sinusoidally modulated over time. However, when tagging coloured objects, it is better to modulate a feature that explicitly represents luminance to ensure that modulation targets luminance directly without unintentionally altering colour balance. This can easily be achieved by converting the RGB colour code to a perceptually uniform colour space, such as CIELAB, which includes a luminance dimension. This luminance component can then be multiplied with a sinusoidal envelope to produce the time-varying tagging amplitude. Finally, these scaled values can be converted back to RGB for drawing the stimulus.

Lastly, it should be noted that tagging colour affects the available dynamic tagging range of luminance. Tagging from 0% luminance (black) to 100% luminance (white) produces a grey tag. Since the luminance of any other colour falls somewhere within this range, tagging other colours reduces the fraction of the luminance range that is sinusoidally modulated. The more perceptually distinct colours (that are equivalently tagged) are required, the lower this possible range gets. Thus, tagging several colours equally comes at some cost to the luminance range, which then translates into a cost to the tagging amplitude in the neural response (Spaak et al., 2024).

### 3.4 Best practices to avoid visibility of the tagging

In addition to using a high tagging frequency (>60 Hz), there are other parameters that heavily influence how “invisible” the tagging is. Consider a tagged patch of background that is beyond an individual’s critical flicker–fusion threshold, in that the luminance oscillation itself is invisible. Since this stimulus is presented against a static background, very abrupt shifts of luminance are produced at the border of the tagged region when the tagging cycle is at extreme values (grey background – white tagging area, grey background – black tagging area). Over time, this is not a concern since the luminance changes are too fast to be perceived. However, during eye movements, the invisibility of the tag is compromised. A faint boundary becomes momentarily visible around the tagged region, likely due to saccadic suppression (Krekelberg, 2010). One simple option to reduce this boundary detection is to place a visible outline around the tagged area, since this tagging boundary is then masked by an actual physical boundary. However, if the goal is to invisibly tag an area that is perceptually indistinguishable from a static background, then this is not an option. In that case, a transparency mask (Minarik et al., 2023) can be applied to the edges of the tagged region to produce a smooth edge that is not detected as a clear boundary between tagged and untagged regions. Alternatively, if the boundary between tagged and untagged regions coincides with the boundary of a visible object, tagging is much less readily perceived. There are also other factors to consider beyond the tagging boundary. The tagging is more easily observed in the higher parts of the luminance range, and in peripheral as opposed to foveal locations. Thus, if using tags in the periphery, the other factors mentioned here will require more attention. Lastly, to reduce visibility resulting from a sudden tagging onset or offset, the tagging may be ramped up or ramped down at the ends of the tagging interval (e.g., 200 ms ramp-up and ramp-down periods as used by Minarik et al., 2023; but we have also used periods as low as 50 ms).

Regardless of such precautions, it is ideal to empirically confirm the luminance modulation being imperceptible. This can either be done beforehand by means of a pilot experiment, or alternatively by informing the participants in advance that they may see certain flickers or glitches during the experiment and conducting an appropriate questionnaire afterwards to see whether they perceived any such effects (Arora, Gayet, et al., 2025; Drijvers et al., 2021; Pan et al., 2021; Seijdel et al., 2024).

### 3.5 Alternative forms of tagging

In this perspective, our suggestions and discussions of RIFT focus on periodic stimulation at specific frequencies, using frequency alone as a channel through which separate tags can be achieved. This approach is the standard taken in almost all existing RIFT work (see Table 1). However, there are also alternative tagging approaches, varying in how extensively they have already been explored in combination with RIFT.

#### 3.5.1 Phase tagging

One alternative involves a different feature of oscillations: phase. Instead of creating only one channel of information for a given tagging frequency, by modulating phase it is possible to achieve multiple channels of information (multiple tags) per tagging frequency (Spaak et al., 2024). This is achieved by tagging separate stimuli or locations at the same frequency, but at some phase offset that is maintained consistently over time and/or trials. This consistency allows the neural responses at that frequency to later be disentangled and attributed to the uniquely phase-locked tags. Though unique frequency tags have separate response profiles and thus cannot be compared with each other and must be counterbalanced (see Section 3.2.2), two phase tags at the same frequency can be directly compared. Given that the frequency range within which RIFT is possible is rather small (see Section 3.1), this approach also offers a promising avenue for maximizing the number of RIFT tags that can be used simultaneously.

#### 3.5.2 Broadband and noise tagging

In broadband tagging, as opposed to stimulating at one specific frequency, the stimulation used contains periodic components at a range of frequencies (e.g., 55–75 Hz broadband tagging as used in Ferrante et al., 2023). A similar alternative comes from the Brain–Computer Interface (BCI) literature in the form of Code-Modulated Visual Evoked Potentials (c-VEPs), also referred to as noise tagging (Martínez-Cagigal et al., 2021). The central idea here is the modulation of luminance using a fully random or pseudorandom temporal sequence instead of a sinusoidal sequence or a sequence limited to a small frequency range. The strength of the neural response to this signal can then be recovered by correlating the M/EEG signal over time with this luminance sequence. One advantage of these approaches over frequency tagging is that the aperiodic luminance sequence allows for an estimation of transmission delay of visual processing, for example, by means of cross-correlating the luminance sequence with the EEG signal; with frequency tagging, the periodicity of the tagging signal prevents this. c-VEPs, like SSVEPs, form an already established field of visual stimulation. However, the advent of RIFT offers a promising future direction: using devices with high-frequency refresh rates allows for these random luminance sequences to be high-pass filtered at 60 Hz prior to display, resulting in random sequences that are compatible with c-VEP but also invisible.

### 3.6 Intermodulation frequencies and auditory tagging

As was briefly outlined in the introduction, RIFT can also be utilized to examine the interaction between two signals, such as audio-visual inputs, by examining intermodulation (IM) frequencies (Drijvers et al., 2021; Hustá et al., 2025; Seijdel et al., 2024). The IM frequency results from the nonlinear interaction of the base audio and visual tagging frequencies and peaks at the difference and sum of the two signals interacting (f2 ± f1; e.g., Regan et al., 1995). The power at the IM frequency is thought to reflect the strength of nonlinear interaction between the representations of the two tagged stimuli. When tagging with frequencies above 60 Hz, the IM component at f2 + f1 exceeds 100 Hz and is virtually undetectable in the M/EEG signal. Consequently, previous studies have focused on analyzing the IM frequency at f2−f1 (Drijvers et al., 2021; Hustá et al., 2025; Seijdel et al., 2024), which is more reliably measurable, but falls within the range of endogenous oscillations. This leads to additional considerations for analyzing IM frequencies. It is best when the oscillatory results for a paradigm are known (such as the established behaviour of alpha oscillations in visual attention paradigms), so the overlap between the endogenous effects and the (chosen) IM frequency can be avoided. This further constrains the selection of the main tagging frequencies. Considering that with certain paradigms, oscillatory effects are difficult to avoid, using a condition without tagging (i.e., tagging baseline) is always advised (see Section 4.2). Importantly, although tagging responses at the carrier frequencies typically decrease with increasing tagging frequency, unpublished data suggest that this negative frequency–amplitude relationship does not necessarily extend to lower-frequency intermodulation (IM) responses. That is, it is not yet clear how the tagging strength at two frequencies relates to the strength of their intermodulation frequency in general. Because IM components are smaller in magnitude than the main tagging peaks, their detectability depends critically on the frequency at which they manifest. This implies that researchers should select tagging frequencies to place the IM response at a neurophysiologically optimal frequency, without being strictly constrained by the reduced amplitude of higher-frequency carrier responses.

When tagging auditory stimuli to study the interaction between auditory and visually tagged stimuli, there are additional considerations beyond those of visual tagging alone. In particular, the duration of the auditory stimulus is critical: short audio segments may not provide sufficient time for reliable frequency tagging. As a result, auditory tagging has often been restricted to longer words (>700 ms). Moreover, the tagging should be applied offline to the audio stimuli before the experiment, so that tagged files can be presented directly during data acquisition and potential timing inaccuracies are avoided. Finally, the perceptual detectability of the modulation should be evaluated. For instance, Drijvers et al. (2021) showed in a pretest that amplitude modulation at 61 Hz did not impair speech intelligibility in clear speech conditions, indicating that high-frequency tagging can be applied without compromising stimulus clarity. We briefly note these caveats here for completeness, but a detailed discussion of auditory tagging lies outside the scope of this paper.

## 4 Analysis Considerations

### 4.1 Common analysis methods

This section outlines common techniques for quantifying the RIFT response. First, we describe two ways of computing the spectral content of the M/EEG signal on each trial. These include **power** as measured by the traditional **Fourier Transform**, as well as the **filter-Hilbert Transform** (filter-HT) approach. Then, we describe how phase-consistency across trials is commonly leveraged by computing (inter-trial) **coherence**.

All techniques listed here are used to measure the magnitude of neural activity at specific frequencies induced by RIFT and to assess the stability and intensity of these responses across trials, conditions, or participants. They all operate on data that have been segmented into epochs after or during preprocessing, often around the onset of the tagging. Which technique to use for estimation of spectral coefficients (FFT versus filter-HT), and for subsequently quantifying the RIFT response (power, amplitude, or (inter-trial) coherence) depends on the exact nature of the experimental design. In general, coherence-like measures provide higher signal-to-noise ratio, so are preferred when aggregating across trials is not a problem. When single-trial estimates are necessary, power or amplitude can be used.

**Fig. 6.**
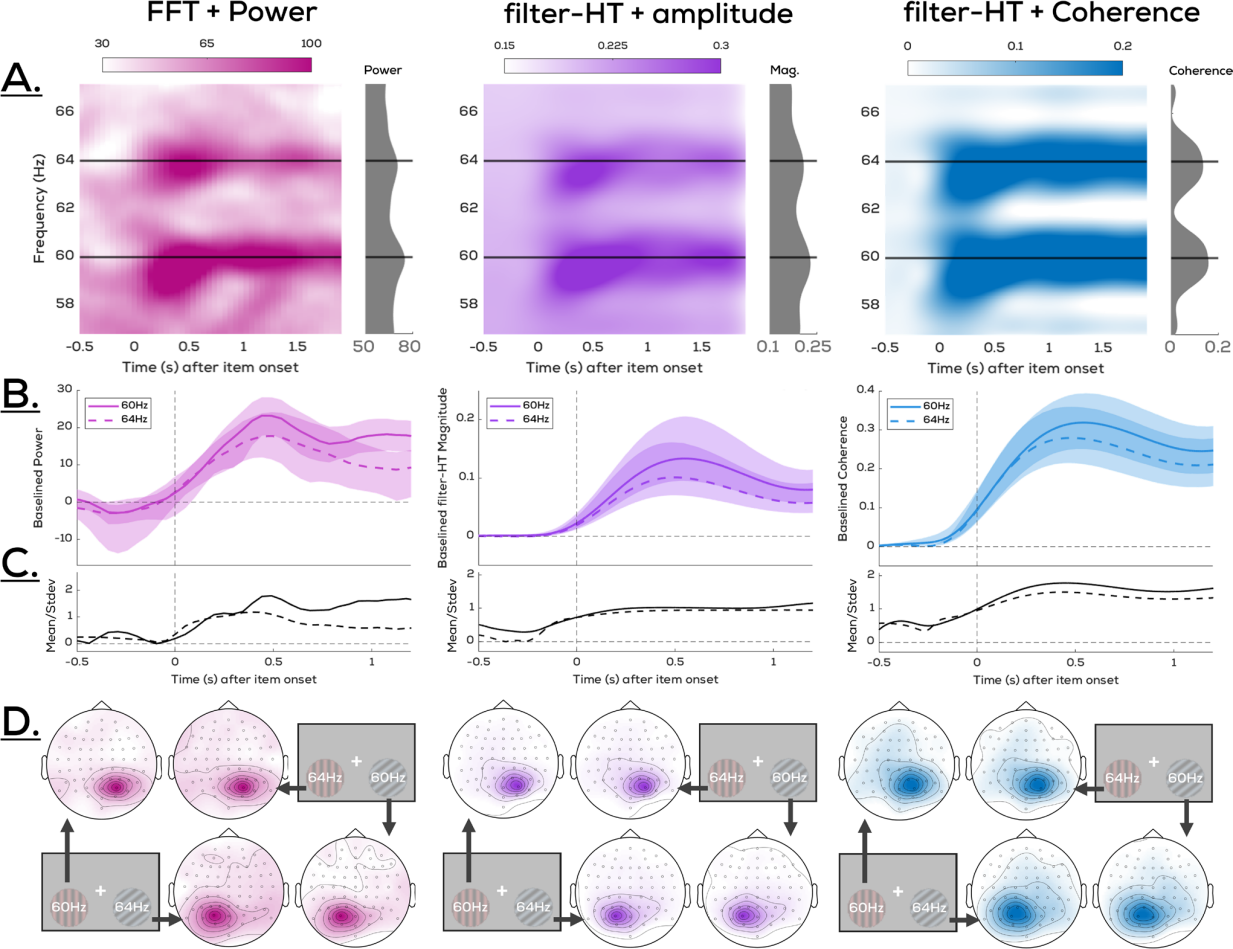
Overview of tagging response as measured by various techniques. (A) Spectrograms, (B, C) traces, and (D) topoplots providing an overview of the RIFT response as measured by (left to right:) trial-averaged FFT-derived power, trial-averaged filter-HT amplitudes, and filter-HT-derived coherence. Shaded regions in (B) represent 95% bootstrapped confidence intervals across participants. Traces in (C) reflect the variance-normalized response strength (i.e., absolute ratio of the mean and standard deviation across participants) for each trace shown in (B). Data averaged across 24 participants from Dataset 1. For filter-HT-based analysis (used here for filter-HT amplitudes and coherence), we used a bandpass width of 2 Hz centred at the frequency of interest for Figure 6A and C, and 3.8 Hz for Figure 6B. For FFT-based analysis (used here for Power), we computed FFTs at a resolution of 0.1 Hz using sliding windows of 1 second duration with 95% overlap.

We subsequently provide an overview of the RIFT response as measured through each of these techniques (Fig. 6). We refrain here from a thorough quantitative comparison of techniques since each involves a range of parameter choices. An in-depth exploration of these parameter spaces is both out of the scope of this document and likely not that impactful for the final takeaways (Bruns, 2004). We also refrain here from covering more specialized techniques that have not yet been combined with RIFT (e.g., wavelet-transforms, superlets; Moca et al., 2021), but could be interesting avenues for future research. Here, we simply offer examples of common tools used to analyze the RIFT response and their outcomes.

#### 4.1.1 Fourier Transform

Power at the tagging frequencies can be computed by converting the time–domain data into the frequency–domain using a Fast Fourier Transform (FFT). The time–frequency trade-off must be considered here: for example, if aiming to achieve 1 Hz frequency resolution, a window length of 1000 ms is required.

Conversion to the frequency domain for regular M/EEG analysis is usually accompanied by tapering of the signal to reduce spectral leakage, that is, the spreading of energy from one frequency to others. Hanning or Hamming windows provide strong attenuation of frequencies far away from the frequency-of-interest (i.e., low side lobes), which is desirable in standard M/EEG analyses. However, based on our findings, boxcar tapers (i.e., essentially not tapering at all) are more effective for capturing tagging responses, due to the narrow main lobe of the boxcar taper.

If multiple frequency tags are present in the signal, ensure that unique tagging frequencies are spaced at least N Hz apart for a 1/N second analysis window (e.g., at least 5 Hz apart for a 0.2 second analysis window, or at least 2 Hz apart for a 0.5 second analysis window) to avoid spectral overlap (or, see alternative suggestion using phase randomization in Section 3.2.1).

There are two options for computing power using the FFT approach. The first is to apply an FFT to individual trials, and then average the resulting spectra across trials. Alternatively, the M/EEG response can first be averaged over all trials (in a given condition), and then an FFT can be applied on the resulting average signal. While the second option also utilizes phase information (i.e., spectral information is only preserved in the average signal if the tagging response is phase-locked across trials), it prevents the use of trial-wise correlations with the measured RIFT response.

#### 4.1.2 Filter-Hilbert Transform

An alternative spectral transformation to the FFT is the filter-Hilbert Transform (filter-HT). The Hilbert Transform is only practically interpretable when applied on monocomponent signals, that is, those produced by a single periodic source. Thus, when computing the HT, an M/EEG signal must first be bandpass filtered at the frequency of interest. For example, filtering a 60 Hz tagging response between 58 Hz and 62 Hz. This can then be referred to as the filter-HT approach. Bandpass filtering requires the selection of a bandpass width parameter. Narrower filter widths reduce the contribution of neighbouring frequencies and noise but provide poorer temporal resolution. Ideally finite impulse response (FIR) filters are used. The advantages over infinite impulse response (IIR) filters include a better-defined passband and zero phase distortion (Widmann et al., 2015). See Section 3.2.1 for an overview of how this parameter choice matters when multiple tags are used.

#### 4.1.3 Coherence

Coherence measures the synchronization between M/EEG signals at specific frequencies and a reference signal, typically a pure sine wave at the tagging frequency or a photodiode recording. In addition to relying on oscillatory amplitude, coherence also relies on how phase locked these oscillations are across a set of trials.

To perform coherence analysis, a reference signal, such as a sine wave matching the tagging frequency, is generated to match the duration and sampling rate of the epochs. If the exact same reference signal (identical frequency and phase) is utilized across trials, the resulting coherence is equivalent to intertrial coherence (ITC). Alternatively, some studies use the photodiode measurements from the on-screen tagging as a reference signal (Duecker et al., 2021; Pan et al., 2021), as this measure provides the ground truth even when encountering imprecisions in the tagging response (e.g., due to missed frames), which would be missed by the sine wave approach or by Fourier transform-based approaches. In this way, coherence offers options beyond, for example, averaging the signal across all trials and then performing a Fourier transform on the average.

Equation (1) provides a measure of how consistently the brain’s activity synchronizes with the tagged stimulus, resulting in a coherence estimate at time t.

$$\text{coh}\left(\text{t}\right)=\frac{\left|{\text{G}}_{\text{xy}}{\left(\text{t}\right)}^{2}\right|}{{\text{G}}_{\text{xx}}\left(\text{t}\right)\;{\text{G}}_{\text{yy}}\left(\text{t}\right)}.$$ (1)

In Equation (1), *G_xy_* is the cross-spectral density of the M/EEG signal and the reference signal, measuring how much the signals oscillate together. *G_xx_* and *G_yy_* are their auto-spectral densities, measuring the strength of oscillations in each signal individually. These spectral densities can be computed in different ways, for example, through an FFT or through a filter-HT, as described above. High coherence implies a consistent response to the tagging frequency. Coherence is computed across time and/or trials. The RIFT literature describes this computation in more detail (Arora, Gayet, et al., 2025; Pan et al., 2021; Spaak et al., 2024).

Comparisons across experimental conditions can be made by computing coherence separately on groups of trials corresponding to specific conditions (rather than on individual trials), and contrasting the resulting coherence traces. Condition-wise coherence can in this manner be computed per channel, per tagging frequency, and per participant.

Coherence is particularly advantageous compared with power because it considers not only the amplitude of oscillatory activity but also the consistency of its phase alignment with the stimulus across trials (figure 3 in Spaak et al., 2024). Consequently, coherence provides greater sensitivity for detecting reliable neural responses at the tagging frequency, even if spectral noise at the frequency of interest is present.

### 4.2 Baselines

For all experiments, we recommend a traditional baseline where no stimuli are presented, tagged or otherwise. This offers an additional SNR metric to demonstrate the strength of the tagged response by comparison with a no-tagging period. However, depending on the research question and its potential confounds, different experiments may require different additional baselines. In cases where cognitive (e.g., attentional) modulations of the RIFT response could be positive (e.g., enhancement) or negative (e.g., suppression), a “tagging baseline” may also be of use. That is, a period of time where the tagged stimuli are presented but without the experimental manipulation of interest. This then provides a base amplitude of tagging compared with which modulations can be then observed as positive or negative. Without such a tagging baseline, even if it is possible to compare different tagging conditions to each other, it may not be possible to tell whether a particular condition reflects a suppression or enhancement of the RIFT response. Other experiments may warrant the use of a “cognitive baseline”, where experimental stimuli are presented without tagging. This can help identify whether and at which frequencies oscillatory effects arise independently of tagging. This baseline is especially critical when analyzing IM frequencies that overlap with lower-frequency bands.

### 4.3 Channel selection

#### 4.3.1 The RIFT topography differs based on tagging location

All analysis techniques described above in Section 4.1 are computed independently for all channels. As is the case with any M/EEG analysis, channel selection is then an important step when analyzing the RIFT response. This is especially true here since the topography of the RIFT response varies based on the tagging location, that is, the same tag may evoke different response patterns across channels in trials with different tagging locations. This is evident from the topographies in Figure 6C, where lateralized peaks are seen depending on which hemisphere the tagged stimulus is displayed in. Channels can either be selected separately for each location at which tags were displayed (but, importantly, still blind to the experimental conditions of interest; Wang et al., 2026), or, first the tagging amplitude across different location presentations can be averaged to produce a general tagging response topography from which channels can then be selected (Arora, Gayet, et al., 2025; Bouwkamp et al., 2025). The former would allow for a stronger response since channel selection can then reflect the unique topographies across different locations of presentation; however, the latter does not require splitting trials into various bins which can negatively affect statistical power and SNR.

**Fig. 7.**
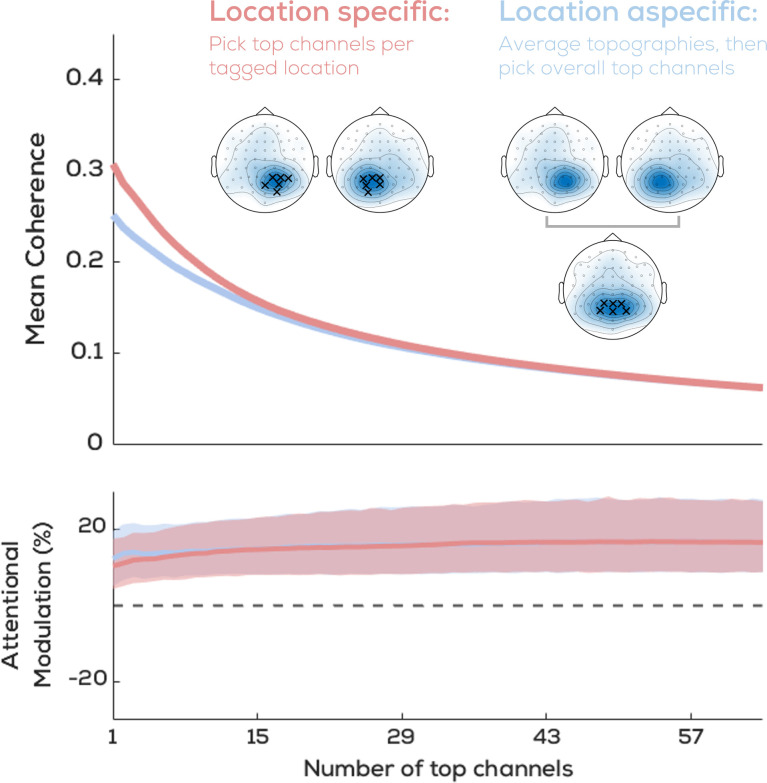
Top–down RIFT modulation is robust across channel selection choices. The attentional modulation of RIFT (as measured across 24 participants from Dataset 1) is independent of the sequence of channel selection and averaging, as well as the number of top channels selected. **Top:** Mean coherence amplitude across participants and **Bottom:** relative attentional modulation of this coherence amplitude as a function of the number of best (highest overall amplitude) channels selected.

Here, we show using Dataset 1 that there is very little difference between the two procedures, in terms of both the resulting RIFT response obtained and the modulation (here by means of covert attentional shifts) to this response (Fig. 7 blue vs. pink). This conveys that it may not be necessary to split trials into conditions based on where the tagging was displayed, retaining higher SNR. Dataset 1 included two different tagging locations each at six degrees of visual angle (dva) horizontal eccentricity.

#### 4.3.2 Number of channels selected does not affect the top–down RIFT modulation

Since slight differences in the exact topographies can be expected across participants (see Arora, Gayet, et al., 2025, for an overview of topographies across 72 participants), most studies conduct a participant-wise selection of channels. This can be done either through a participant-wise selection of “n” channels that show the strongest tagging response, or a selection of all channels that show a significant difference in tagging response from baseline. In case a channel-average referencing procedure is implemented during preprocessing, the former option is preferred. Making a selection of top channels should ideally be done using an independent dataset, for example, a tagging baseline (see Section 4.2) where the tagging is presented without the experimental manipulation of interest.

Here, we show that attentional modulations to RIFT are independent of the exact number of channels selected. We looked at the effect of how many channels are selected on coherence and its attentional modulation as measured from Dataset 1 (Fig. 7). Although average coherence decreases as more channels are selected, the relative top–down modulation of this coherence (% change with attention in/out) is unaffected regardless of how many channels are selected, meaning that the exact number of channels selected is not as relevant. Note that here we assess these channel-selection-related criteria using 64-channel EEG data. However, it is possible that the constraints on channel selection and the differences between approaches assessed here may matter more when using systems with fewer channels.

Another option is to circumvent concerns with number of channels entirely by using tools that meaningfully combine the signal across all channels. This could be with the use of spatial filters such as Rhythmic Entrainment Source Separation (RESS; Cohen & Gulbinaite, 2017) that weigh the signal of each channel based on their individual contributions. Alternatively, this could be with source reconstruction tools such as Dynamic Imaging of Coherent Sources (DICS; Gross et al., 2001) that provide a measure of the neural response from an anatomically informed virtual sensor.

#### 4.3.3 Additional considerations for MEG

With MEG there is an additional consideration, namely that of whether to use magnetometers or gradiometers. Previous work showed that the two measures are largely comparable; however, magnetometers compared with planar gradiometers had stronger sensitivity to tagging (Minarik et al., 2023). A majority of the current studies use axial gradiometers, which some convert to planar gradiometers (e.g., Bouwkamp et al., 2025; Drijvers et al., 2021; Seijdel et al., 2024; Zhigalov et al., 2019).

A further concern with MEG, compared with EEG, is that the sensor topographies are likely more heterogeneous across participants (i.e., EEG is strongly spatially low-pass filtered). Therefore, more care is needed for channel selection in MEG than in EEG. The case for (anatomical and/or signal-driven) spatial filtering may thus be stronger in MEG than in EEG.

### 4.4 Does the RIFT signal vary much across participants? Where does the variation come from?

**Fig. 8.**
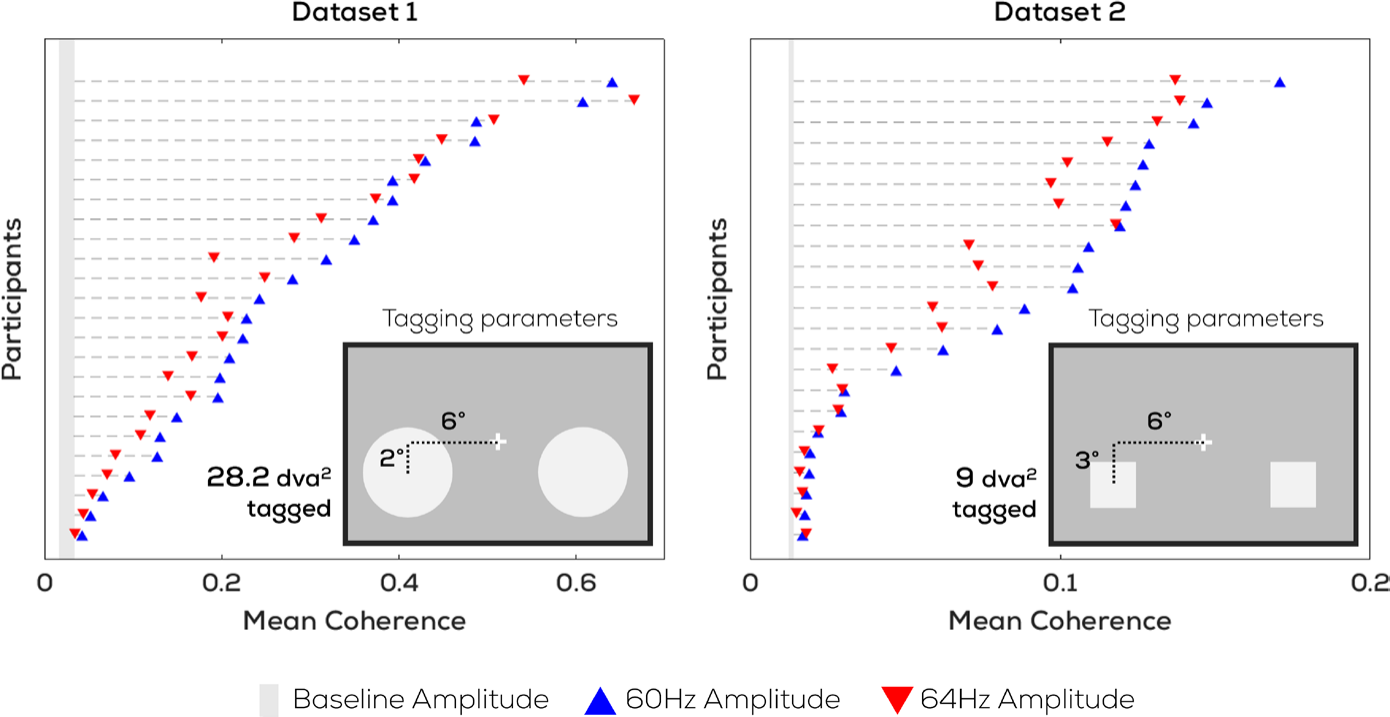
Spread of 60 Hz and 64 Hz coherence across participants in Datasets 1 and 2. Coherence amplitudes from two tagged frequencies compared with non-tagged frequency baseline for (A) Dataset 1 (B) Dataset 2. Insets show tagged stimulus size and eccentricity. Dataset 2 shows a larger proportion of participants with a tagging response minimally distinguishable from baseline, most likely due to smaller tagged area.

With RIFT, we operate close to the threshold of stimulation that does not produce a measurable tagging response in the M/EEG signal (>~72 Hz as discussed in Section 3.1). Even when operating at a feasible tagging frequency, making stimuli smaller, more peripheral, or using less of the dynamic luminance range may eliminate a measurable tagging response. Importantly, each participant’s tagging amplitude drop-off may scale differently with any of these factors (Minarik et al., 2023). It is worth highlighting this because in the authors’ experience, a tagging response is not visible in every participant with every tagged stimulus design. In some designs, only two-thirds of the participants show viable peaks at the tagging frequencies compared with non-tagged frequencies (see, e.g., Pan et al., 2021), even when using more sensitive analysis methods such as coherence. Here, we visualize the spread of the tagging response across participants in two experiments (Fig. 8), one with a relatively large tagged area showing a response in almost all participants (Dataset 1) and the other with a relatively smaller tagged area showing a response in roughly three-quarters of participants (Dataset 2).

**Fig. 9.**
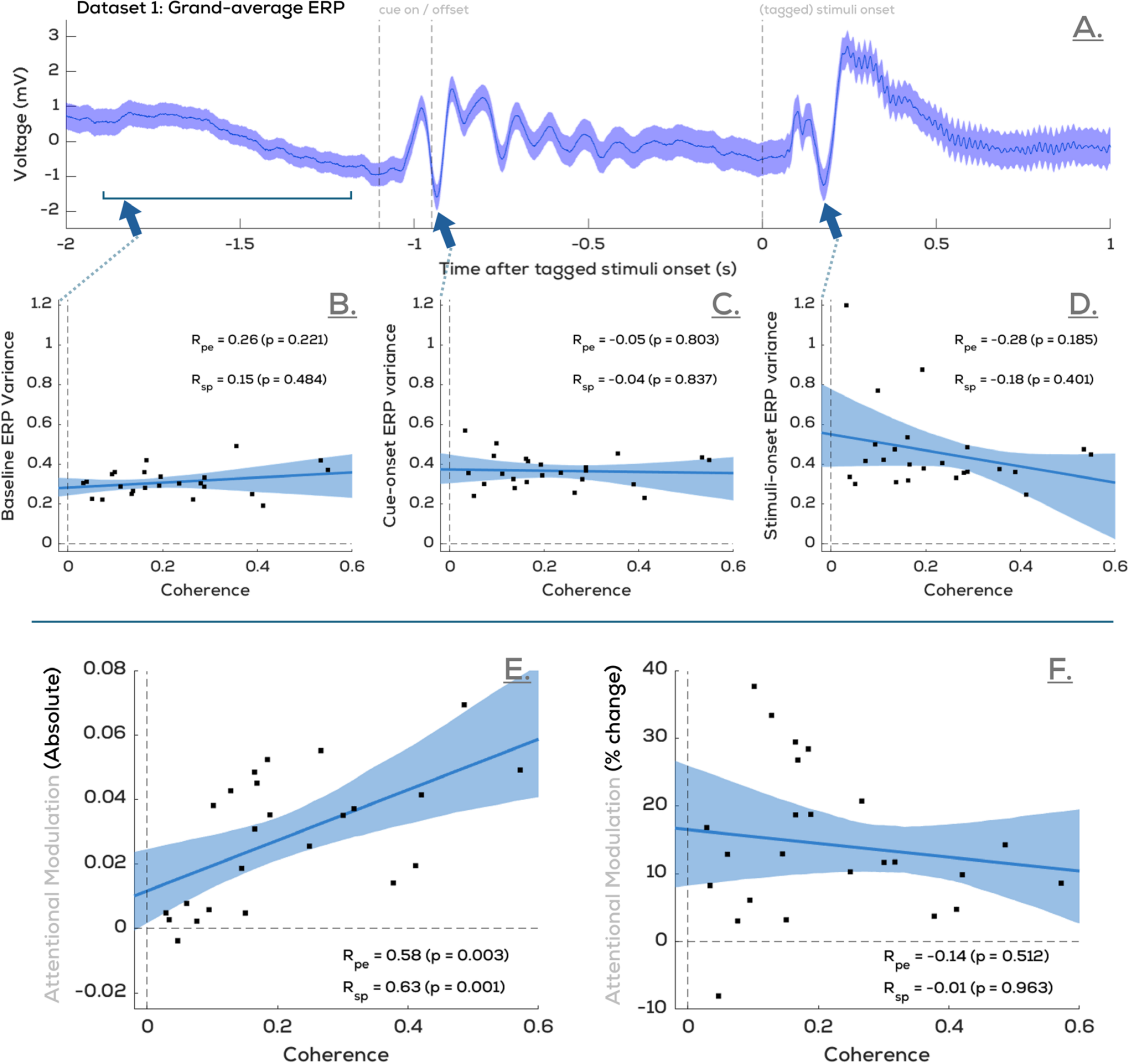
Coherence amplitude is not correlated with trial-wise ERP variation across participants. (A) ERP averaged across participants and top channels similarly to coherence, shaded areas represent mean trial-wise SEM. Pearson (R_pe_) and Spearman (R_sp_) correlations between participant-wise coherence amplitude and trial-wise SEM of (B) baseline (-1.8 to -1.1 seconds), (C) cue ERP (-0.97 seconds), and (D) stimuli ERP (0.17 seconds). Coherence amplitude is correlated with absolute, but not relative, attention modulation. Pearson (R_pe_) and Spearman (R_sp_) correlations between coherence amplitude and (E) absolute attentional modulation of coherence (F) relative attentional modulation of coherence. Coherence and attentional modulation from Dataset 1 averaged in the interval with a significant attentional modulation at the group level (0.28–1.14 seconds after tagging onset). Data from the 24 participants of Dataset 1.

Even without participants who can be qualitatively labelled as “unresponsive” to the tagging, there is a lot of variability in the strength of the response of Dataset 1. We conducted further analyses in an attempt to identify the source of this variability. One possibility is that this variability only reflects standard collection noise, since any measure of frequency amplitude or phase would be negatively impacted in participants with noisier signals. Such noise would also be visible as variability in the raw voltage recordings. We correlated the participant-wise variability in the raw voltage (trial-wise standard error in event-locked ERPs) to coherence. Despite picking several points of time along the trial to use for the voltage variability measure, we observed no correlation between signal noise (ERP variability) and coherence (Fig. 9A–D). That is, participant variability in the tagging response is not driven by overall noise in the data.

Next, we wanted to see whether this variability in the tagging amplitude affects the attentional modulations that can then be observed in the tag. With Dataset 1, for example, this effect of interest comes from increased covert attention at the tagging location to encode an item presented there. We compared the participant-wise coherence with the attentional modulation of this signal (i.e., the boost in the RIFT response from covertly attending the tagged location) using Dataset 1. Naturally, having a higher overall coherence was strongly linked to a higher *absolute* attentional effect (Fig. 9E). However, interestingly, when looking at the *relative* attentional effect (i.e., the cognitive effect as a fraction of the overall coherence), there was no benefit afforded by higher overall coherence amplitudes (Fig. 9F). That is, the effect observed in Figure 9E is caused only by linear scaling of the amplitude modulation. Thus, variability in tagging strength across participants may not be of consequence to the experimental manipulation of interest, provided that a viable tagging peak is observed.

### 4.5 Spontaneous eye movements around fixation do not impact the RIFT response

Most studies on covert attention, or shifts of spatial attention, require participants to fixate the centre of the screen while some stimuli of interest are presented in the periphery (Posner, 1980). Since in the visual domain RIFT often acts as a tracker of spatial attention, this is also the design of many RIFT studies (Arora, Gayet, et al., 2025; Bouwkamp et al., 2025; Ferrante et al., 2023; Seijdel et al., 2024; Zhigalov et al., 2019). The fixation requirement is frequently controlled with an eye tracker, so that trials containing large saccades can be identified and excluded. But measures like RIFT are subject to an additional eye movement-related concern: visual stimuli close to the fovea are processed much more strongly than those in the periphery. The absence of large saccades towards a tagged stimulus or location does not eliminate the presence of small deviations in gaze position (<1 dva) that might nonetheless bring the tag closer or further away from the fovea. Does variability in gaze position at these small scales consistently drive the RIFT response?

This question has previously been addressed at the trial level when linked to consistent attentional modulations of gaze position through microsaccadic action (Arora, Gayet, et al., 2025), and we recommend researchers to conduct similar trial-level tests when using RIFT in designs where eye movements can be a potential confound. Here, we present a more sensitive analysis in which we conducted a timepoint-by-timepoint comparison of gaze position and the RIFT amplitude of two peripheral tags (one at 60 Hz and the other at 64 Hz) on the screen from Dataset 1. The trials from Dataset 1 consisted of two tagged stimuli slightly below left and right of fixation.

**Fig. 10.**
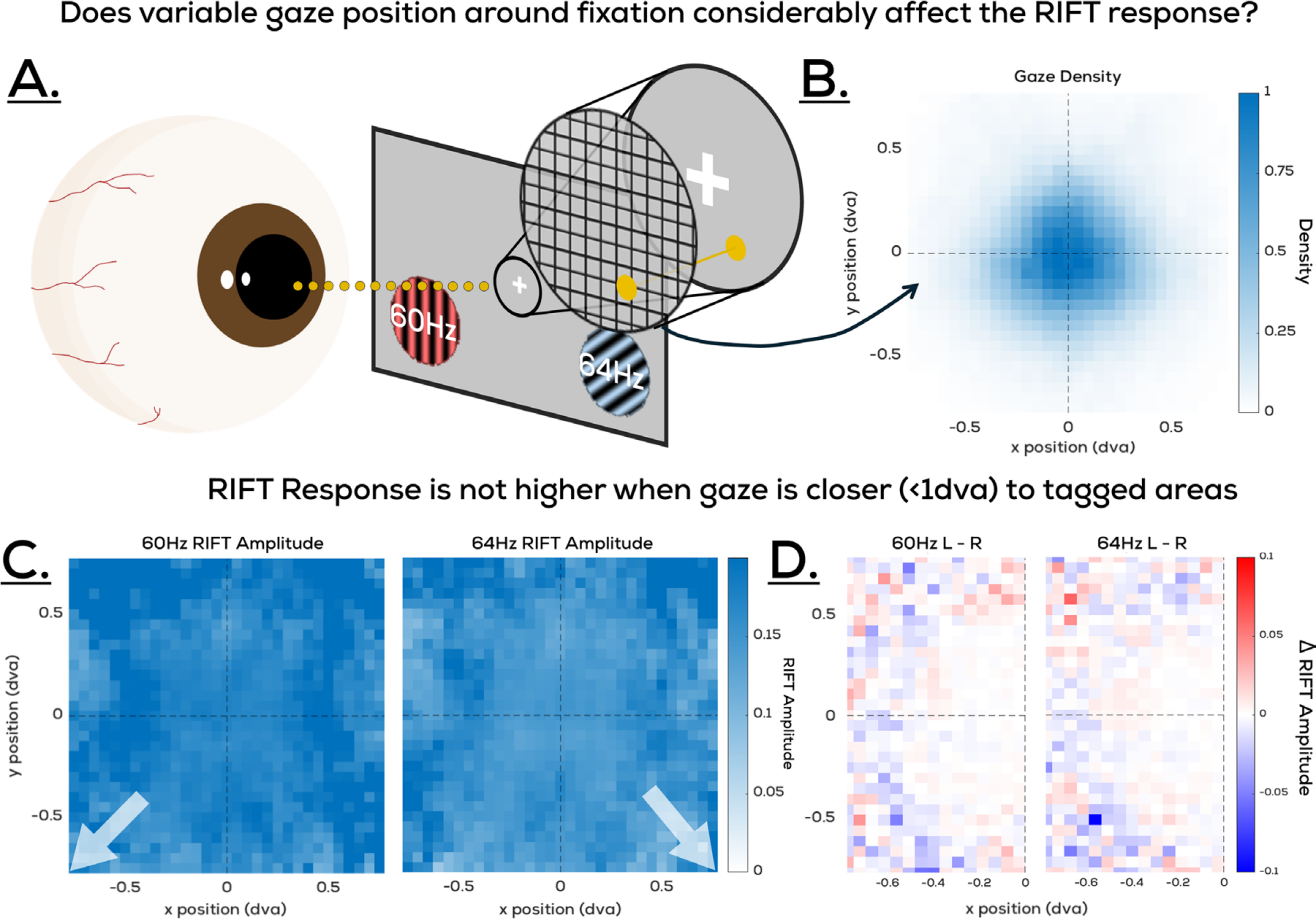
Minor gaze deviations (<0.75 dva) around fixation do not correlate with the RIFT response. (A) Dataset 1 contained one 60 Hz and one 64 Hz tagged stimulus on each trial (B) Density plot of gaze position averaged over participants and the 1 second interval of item presentation. (C) Mean 60 Hz and 64 Hz tagging signal when gaze position was in the corresponding bins. Arrows point towards the location of corresponding tagged stimuli. (D) Lateralization in tagging signal; a cluster permutation test showed no significant clusters, that is, small gaze deviations towards the left vs. the right do not enhance the tagged response of the stimulus on the left vs. the right.

First, we computed participant-wise gaze densities within a square extending 0.75 dva on either side horizontally and vertically from fixation (Fig. 10B). During this task, participants had been instructed to maintain fixation, and gaze was present within this square for 96.7% of the duration used. Then, we conducted the following analysis for each binned section of the gaze density plot (bin width 0.05 dva). We isolated all the timepoints during which gaze was within that particular bin. We then averaged the RIFT amplitude over all these timepoints. Thus, we obtained a measure of the mean RIFT amplitude from when participants’ gaze position was localized within each bin (Fig. 10C). We next tested whether gaze deviation away from fixation and towards the tagged stimuli locations influenced the RIFT amplitude. The lateralization plots testing this (left minus right; averaged across participants; Fig. 10D) show no consistently positive difference for 60 Hz or negative difference for 64 Hz. This was also statistically confirmed with a 2D cluster-based permutation test (Maris & Oostenveld, 2007).

Thus, small fixational eye movements (<0.75 dva) do not meaningfully drive the RIFT response. This confirms that an enhancement of the RIFT response measured in such cognitive tasks can reflect a genuine top–down modulation of the responsiveness to the tagged location or stimulus, rather than simply being a correlate of changes in foveation.

## 5 Interpreting the RIFT Response

RIFT utilizes a relatively well-understood property of the visual system: its responsiveness to changes in luminance. Unlike traditional SSVEP, it achieves this without being consciously perceived. Since conscious perception is associated with more downstream areas of the visual hierarchy, this implies that the response to RIFT stimulation is limited to upstream visual areas, that is, early visual cortex. This selectivity is part of what makes RIFT attractive: conventional stimuli unavoidably elicit responses from the whole visual system, but this technique allows researchers to non-invasively measure a response that is selectively obtained from an early subset of this system and thus not a direct readout of high-level processing. The well-established attentional modulation of RIFT responses, therefore, likely reflects the influence of attentional processing in higher-order regions on the early visual cortex activity.

Where does the boundary lie between the neural activity captured by RIFT and the subsequent downstream processing? At the moment, this question cannot be answered with precision and certainty. The few existing studies that have utilized RIFT do not convey a unanimous answer to this question. Terms such as “cortical excitability” (Zhigalov et al., 2019), or [responses from] the “early visual cortex” (Arora, Gayet, et al., 2025; Drijvers et al., 2021; Pan et al., 2021), “predominantly primary visual cortex” (Duecker et al., 2025), “occipital cortex” (Minarik et al., 2023), have been used to describe the RIFT response, both with and without the explicit mention of regions such as V1 or V2. There is no doubt that the peak RIFT response is in fact localized to relatively early processing areas; its retinotopy and existing MEG source localization work (Drijvers et al., 2021; Duecker et al., 2025; Ferrante et al., 2023; Minarik et al., 2023) are clear evidence of this. However, even within these studies, there are slight variations in regions to which the RIFT response is attributed (e.g., “V1”: (Duecker et al., 2025) “V1/V2”: (Ferrante et al., 2023)), leaving the question of how far exactly the RIFT signal propagates currently open. On the one hand, broadband tagging approaches have not found evidence for tagging responses at multiple distinct phase lags (Ferrante et al., 2023), as high propagation would predict. On the other hand, applying a combination of visual and auditory tagging has given rise to IM frequencies that have been localized beyond regions typically responsive to sensory processing, such as (left) frontotemporal cortex (Drijvers et al., 2021; Husta et al., 2025; Seijdel et al., 2024). This suggests that, intriguingly, [consequences of] RIFT signals may progress to downstream areas of the visual hierarchy, where consequences of their (nonlinear) processing, that is, the IM peaks, are detectable.

Thus, it is currently unclear what the exact spread of the RIFT response within the cortex looks like. A final note, however, is that for several branches of cognition research, the main utility of RIFT is not achieved directly from the section of cortex that RIFT selectively activates, but rather its invisibility. RIFT evokes a response in the brain without being perceived, regardless of *where* in the brain it evokes this response.

## 6 Closing

RIFT provides a powerful new way to study rhythmic cognition. By embedding invisible, high-frequency tagging into stimuli, it offers a clean and continuous marker of neural processing that avoids the perceptual confounds of traditional frequency tagging. As this manual outlines, careful attention to hardware, tagging parameters, and analysis methods is imperative for the success of RIFT experiments. This manual, therefore, serves as a guide for implementing Rapid Invisible Frequency Tagging (RIFT) in cognitive neuroscience research. By outlining detailed protocols and recommendations for setup, experimental design, and analysis (based on both experience and data-derived evidence from multiple laboratories), it provides researchers with the necessary tools to leverage RIFT’s capabilities effectively. With these best practices in place, RIFT opens opportunities to probe attention, perception, memory, and multimodal integration, under naturalistic settings, thus bridging the gap between experimental research and everyday cognitive functions. In sum, we hope that this guide will help establish RIFT as a standard approach for studying the oscillatory dynamics that shape human cognition.

Looking ahead, there is a compelling need for ongoing research aimed at enhancing the accessibility of the required technology, refining the procedures to minimize variability, developing more robust methods for data analysis, and expanding the growing list of theoretical questions that RIFT is used to study. By continuing to develop and apply the RIFT technique in cognitive research, the neuroscience community can push the boundaries of our understanding of brain function and cognition.

## Supplementary Material

Supplementary Material

## Data Availability

We make use of two previously collected datasets to demonstrate common RIFT design and analysis techniques. In Dataset 1 (Arora, Gayet, et al., 2025), 24 participants performed 480 trials each of a working memory task (the “pre-cue” experiment in the corresponding publication). Here, we only make use of the 1 second period of this task when two gratings, tagged at 60 and 64 Hz, were displayed in the lower visual field. In Dataset 2 (Arora, Van der Stigchel, et al., 2025), 23 participants viewed a grid display with 3 grid locations, tagged at 60 Hz, 64 Hz, and 68.5 Hz, respectively. Supplementary Figure S2 provides an overview of the stimulus and tagging parameters for both datasets. Further details are given in the corresponding publications. Example experimental files and useful functions for designing RIFT experiments in combination with the PROPixx projector are given in the following repository: https://osf.io/9mv3e/
