# Supplementary material for: A collaborative guide to Rapid Invisible Frequency Tagging (RIFT): Methods, insights, and recommendations
Source: Imaging Neurosci (Camb). 2026 Jun 17;4:IMAG.a.1273. doi: 10.1162/IMAG.a.1273 (PMC13277780; doi:10.1162/IMAG.a.1273)
Supplement: Supplementary Material [file IMAG.a.1273_supp.pdf]

Supplementary Material

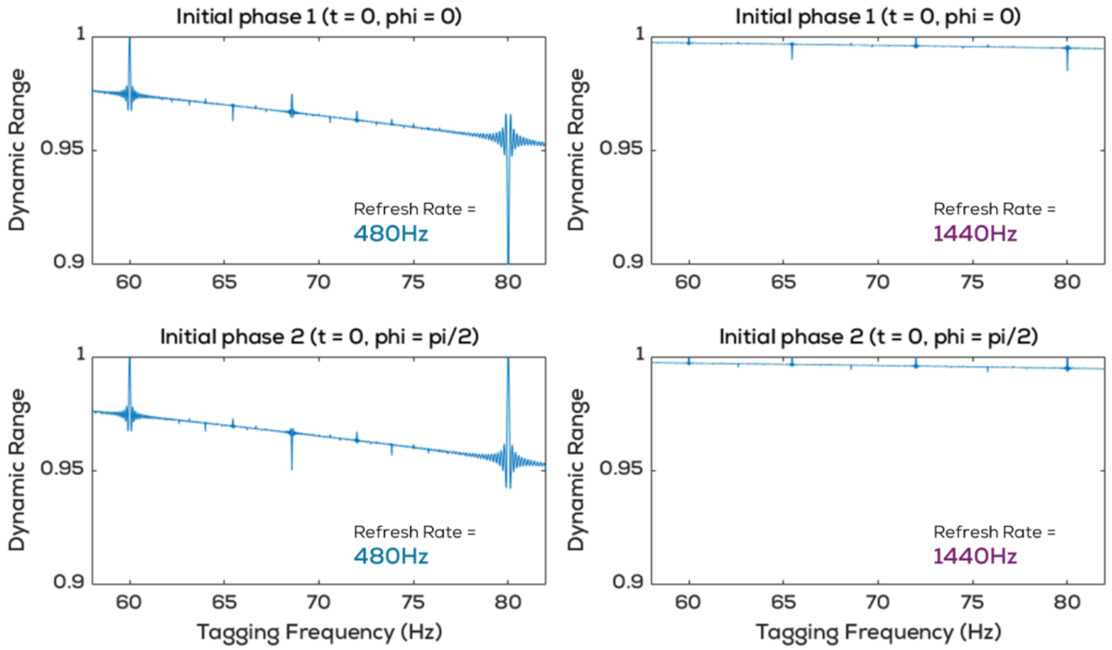

Figure S1: Effect of sampling on dynamic range, as modulated by tagging frequency (x-axis), initial phase (top vs. bottom panel), and refresh rate (left vs. right panel). In the main manuscript, we discuss which frequencies are best-suited for tagging, and that sampling parameters can affect how close to ideal the presented luminance modulation is (Figure 4). Here, we extend the ideas of Figure 4, showing that dynamic range - the proportion of the full luminance range from black to white that is used by the tagging sinusoid - depends on various factors such as the selected tagging frequency, but also the initial phase with which it is sampled Dynamic range is computed as the mean amplitude difference between the high and low peaks of the tagging sinusoid; perfectly sampled peaks and lows produce a dynamic range of 1.

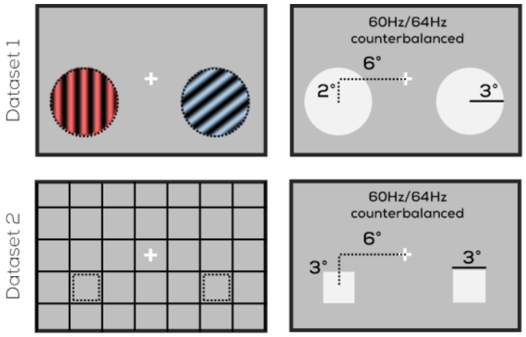

Figure S2. Screen display and tagging parameters for Datasets 1 and 2.
